# Supplementary material for: Characterization of the Volatile Compounds of Onion with Different Fresh-Cut Styles and Storage Temperatures
Source: Foods. 2022 Nov 27;11(23):3829. doi: 10.3390/foods11233829 (PMC9736120; doi:10.3390/foods11233829)
Supplement: Supplementary file 1 [file foods-11-03829-s001.zip › foods-1941952-supplementary.pdf]

Table S1 Volatile sulfur compounds in onions identified by headspace solid-phase microextraction−gas chromatography−mass spectrometry.

No.	Name	Formula	Ion	RI [a]	Relative Content [b]									Identify [b]
					4 °C			20 °C			25 °C			
					S-1	S-2	S-3	S-4	S-5	S-6	S-7	S-8	S-9	
1	Propan-1-thiol	C_3H_8S	76,47,27	857	62.7±3.2a	14.1±0.1b	12.0±0.1b	65.4±1.5a	5.6±0.4c	6.8±0.6c	9.7±0.6c	25.3±0.9d	21.2±1.0d	MS, RI
2	2,4-Dimethylthiophene	C_6H_8S	111, 97, 45	1253	78.5±5.2a	186.0±10.3b	269.7±12.8c	255.1±12.0c	289.0±15.2c	268.1±15.5c	360.0±15.3d	129.6±9.5e	236.8±16.9c	MS, RI
3	Dipropyl disulfide	$C_6H_{14}S_2$	150,108,43	1402	79.0±5.1a	63.9±3.5ab	53.4±4.2b	79.2±4.2a	56.1±6.2b	56.2±4.9b	52.8±4.2b	136.2±6.2c	134.9±9.6c	MS, RI
4	(E)-1-(Prop-1-en-1-yl)-2-propyldisulfane	$C_6H_{12}S_2$	148, 106,41	1425	118.7±5.5a	77.8±6.5b	66.6±5.1c	58.6±3.2d	35.4±1.3e	18.6±0.3f	199.9±6.9g	37.0±1.2e	30.0±1.1e	MS, RI
5	1-Allyl-2-isopropyldisulfane	$C_6H_{12}S_2$	148,106,41	1439	77.8±4.6a	50.5±4.6b	55.5±4.6b	136.9±9.9c	59.2±4.7b	81.0±5a	59.3±5.3b	121.8±6.8c	217.7±9.6d	MS, RI
6	Methyl propyl trisulfide	$C_4H_{10}S_3$	154, 112, 43	1524	55.4±5.0a	83.3±7.5b	26.5±1.6c	94.2±8.6d	89.8±5.2b	75.2±5.7b	118.9±9e	92.5±6.8d	126.1±8.8e	MS, RI
7	2-Mercapto-3,4-dimethyl-2,3-dihydrothiophene	$C_6H_{10}S_2$	113,97,146	1619	183.1±1.2a	470.0±35.2b	882.9±66.3c	797.8±54.6d	854.4±52.6c	925.5±45.7e	1061.6±65.3e	333.9±22.8f	782.8±46d	MS, RI
8	Dipropyl trisulfide	$C_6H_{14}S_3$	182, 75,43	1650	34.5±1.2a	5.8±0.1b	4.4±0.4b	31.7±2.3a	6.3±0.4b	4.6±0.4b	13.5±1c	27.2±2.1d	13.6±1.0c	MS, RI
9	(E)-1-(Prop-1-en-1-yl)-3-propyltrisulfane	$C_6H_{12}S_3$	180, 74,106	1735	31.3±1.0a	23.5±1.2b	23±2b	79.1±2.6c	29.8±1.4b	23.6±1.9b	49.6±2.3av	45.2±2.5ac	57.7±3.6d	MS, RI
10	1-(Methylthio)propyl)-2-propyldisulfane	$C_7H_{16}S_3$	89, 41, 61	1811	34.5±1.2a	7.1±0.6b	4.8±0.1b	48.1±4.2c	10.3±0.6b	6.5±0.4b	22.3±1.6d	14.1±1b	8.8±0.6b	MS, RI
11	1-Methyl-2-(1-(propylthio)propyl)disulfane	$C_7H_{16}S_3$	117, 75,41	1903	111.5±7.6a	86.3±5.5b	118.1±8.6a	214.7±8.8c	39.6±3.1d	19.5±1.2d	121.7±9.9a	12.6±5.5d	10.2±0.7d	MS, RI
12	2-Methoxy-5-methyl-Thiophene	C_6H_8OS	128, 113, 85	1917	58.4±3.2a	13.9±1b	27.1±0.2c	113.6±10.5d	14.2±1.2e	7.0±0.5e	26.4±1.5c	6.7±0.6e	6.3±0.6e	MS, RI
13	Methyl 1-(1-propenylthio)propyl Disulfide	$C_7H_{14}S_3$	115, 73, 81	1969	49.3±2.6a	12.1±0.7b	12.6±0.9b	88.4±3.5c	6.4±0.4d	4.1±0.1d	44.2±3.7a	7.6±0.4b	5.5±0.2b	MS, RI
14	(E)-3,6-diethyl-1,2,4,5-tetrathiane	$C_6H_{12}S_4$	73, 212, 138	2097	23.2±1.9a	46.8±3.2b	32.3±1.9ab	57.9±4.2c	67.8±4.2cd	97.4±4.9d	120.4±9.6e	42.6±3.2b	93.3±8.6d	MS, RI
15	6-Ethyl-4,5,7,8-tetrathiaundecane	$C_9H_{20}S_4$	73,149,43	2153	74.3±5.9a	18.9±1.2b	21.6±1.6b	107.9±8.7c	10.8±1d	6.0±0.5d	78.3±4.9a	15.7±1.2b	9.1±0.8d	MS, RI
16	(Z)- 1-(1-propenyldithio)propyl propyl disulfide	$C_9H_{18}S_4$	73, 105,149	2195	53.8±2.2a	14.8±1.2b	15.1±1.1b	68.0±5.1a	10.6±1.0b	10.4±0.7b	24.7±1.2c	20.9±1.0c	17.8±0.7c	MS, RI
	Total				1126.2±66.6	1174.9±82.4	1625.7±111.5	2296.9±143.9	1585.5±98.9	1610.4±88.3	2363.3±133.1	1069.0±71.7	1771.8±109.8	

[a], **RI** means retention indices;

[b], the relative content of each compound was the ratio of the peak area of each compound in onions stored in different shapes to the peak area obtained in untreated onions (control group). Values (means ± SD, n = 3) with different letters (a, b, c, d, and e) indicate signiffcantly difference at $p \leq 0.05$ (Duncan test) "S-1", "S-2", "S-3" represent the onion bulbs, onion rings, and onion squares stored at 4 °C; "S-4", "S-5", "S-6", represent the onion bulbs, onion rings, and onion squares stored at 20 °C; "S-7", "S-8", and "S-9" represent the onion bulbs onion rings, and onion squares stored at 25 °C.. [b], MS means the compound was identified by mass spectra,
